# Application of different CO_2_ pneumoperitoneum pressure in laparoscopic pyeloplasty for infants with ureteropelvic junction obstruction

**DOI:** 10.3389/fped.2024.1380985

**Published:** 2024-09-23

**Authors:** Yan Peng, Min Zhu, Chunmei Chen

**Affiliations:** Department of Anesthesiology, Children’s Hospital of Nanjing Medical University, Nanjing, Jiangsu, China

**Keywords:** ureteropelvic junction obstruction, laparoscopic pyeloplasty, CO_2_, pneumoperitoneum insufflation, cytokine

## Abstract

**Background:**

Laparoscopic pyeloplasty is a minimally invasive approach for the therapy of infant ureteropelvic junction obstruction (UPJO), reliant on CO_2_ pneumoperitoneum insufflation. While the impact of CO_2_ insufflation on adult and older pediatric populations has been studied, its effects on infants remain less explored.

**Methods:**

This prospective randomized controlled trial included infants with UPJO undergoing laparoscopic pyeloplasty. Patients were allocated to low pneumoperitoneum pressure (LPP, 5 mmHg) or high pneumoperitoneum pressure (HPP, 8 mmHg) groups. Surgical parameters, postoperative complications, acid-base balance, stress markers, inflammatory cytokines, and oxidative stress markers were evaluated and compared.

**Results:**

A total of 116 infants were analyzed. Preoperative characteristics were comparable between LPP and HPP groups. No significant differences in blood loss, operation time, or hospitalization time were observed. Postoperative complications were similar between groups. Acid-base balance analysis revealed a decrease in pH after pneumoperitoneum in both groups, with greater reductions in actual base excess and standard base excess in the HPP group. Stress markers, cytokines, and oxidative stress markers increased postoperatively in both groups, with higher levels in the HPP group.

**Conclusion:**

HPP leads to more pronounced physiological responses, including acid-base alterations, stress reactions, and inflammatory cytokine elevations.

## Introduction

Ureteropelvic junction obstruction (UPJO) is a significant urological condition characterized by a constriction or partial blockage at the point where the renal pelvis connects with the ureter (1, 2). This condition can impede the normal flow of urine from the kidney to the bladder, potentially leading to a spectrum of clinical manifestations and requiring medical intervention (3–5). UPJO can occur due to congenital abnormalities or acquired factors, and its timely diagnosis and management are paramount to preserving renal function and preventing complications (6).

Laparoscopic pyeloplasty stands as a well-established and sophisticated surgical approach in the armamentarium for addressing UPJO (1, 7). This minimally invasive procedure offers a distinct advantage over traditional open surgery, combining precise surgical techniques with reduced morbidity and faster patient recovery times (8, 9). The procedure's small incisions result in reduced tissue trauma, leading to less postoperative pain, shorter hospital stays, and quicker recovery times for the patient (10–12). The minimized risk of wound infections and hernias due to smaller incisions contributes to fewer postoperative complications (13).

Insufflation is a necessary prerequisite for laparoscopic surgery, with CO_2_ currently being the most commonly used insufflation gas in clinical practice (14). Higher CO_2_ insufflation pressures can provide the surgeon with a clear surgical field and a spacious operating area, but they can also impose greater physiological stress on the body, leading to adverse effects (15, 16). Previous research on the impact of CO_2_ insufflation on physiological function has predominantly focused on adults and older children, encompassing a variety of diseases and laparoscopic surgical approaches (15). However, there is a lack of relevant research concerning the infant population. During the infancy period (1–12 months of age), the body's internal regulatory systems are not yet fully developed, rendering them more susceptible to the influence of CO_2_ insufflation during laparoscopic procedures (17, 18). Herein, there is significant clinical importance to investigate the effects of CO_2_ insufflation on the physiological function of infants.

## Methods

### Study design and participants

This study is a prospective randomized controlled trial that enrolled infants with UPJO who were scheduled to undergo laparoscopic pyeloplasty at our hospital. All enrolled patients had unilateral UPJO, without any other complications, and did not have contraindications to laparoscopic surgery such as diffuse peritonitis with intestinal obstruction or abdominal hernias. Informed consent was obtained from the guardians of the patients. Utilizing a random number table, the patients were randomly allocated into two groups: the low pneumoperitoneum pressure (LPP, 5 mmHg) group and the high pneumoperitoneum pressure (HPP, 8 mmHg) group, with 58 cases in each group. The researchers were blinded to the participants’ group allocation. The study was approved by the Ethics Committee of Children's Hospital of Nanjing Medical University. The study was performed in strict accordance with the Declaration of Helsinki, Ethical Principles for Medical Research Involving Human Subjects. The registration number of this study in our hospital is #CTR-2020.568d4.

### Invention

All patients underwent surgery under standardized anesthesia protocols performed by the same surgical team. General anesthesia with standardized endotracheal intubation was administered to all patients, and respiratory parameters were not adjusted during the surgery. The patients were positioned in a lateral decubitus position, tilted at 30 degrees towards the affected side. A two-port technique for laparoscopic pyeloplasty was employed for all cases. At the initiation of surgery, CO_2_ gas was insufflated into the abdominal cavity using a Stryker 1288 insufflator (Stryker, Kalamazoo, MI) at a rate of 1–1.5 L/min. In both groups, insufflation pressures were set to 5 mmHg and 8 mmHg, respectively, and maintained steadily throughout the procedure. At the conclusion of the surgery, all trocars were removed, and the peritoneum was released through the umbilical incision to thoroughly evacuate the CO_2_ gas from the abdominal cavity, thereby concluding the insufflation process.

### Arterial blood parameters

The ‘Danish Radiometer ABL80 Blood Gas Analyzer’ (Radiometer, Copenhagen, Denmark) was employed to measure arterial blood parameters, including pH, arterial partial pressure of carbon dioxide (PaCO_2_), arterial partial pressure of oxygen (PaO_2_), actual base excess (ABE), and standard base excess (SBE), at four distinct time intervals: 5 min before insufflation, 5 min after insufflation, 30 min after insufflation, and 30 min after desufflation. Arterial blood samples were collected during each of these time points.

### Inflammation and stress response

For both groups of patients, venous blood samples of 5 milliliters each were collected at three distinct time points: 5 min before insufflation, upon completion of surgery (during closure of the insufflation incisions), and 24 h postoperatively. These samples were subjected to anticoagulant treatment and subsequently placed in a low-temperature centrifuge at a speed of 3,000 rpm for 15 min. The resulting supernatant was extracted and stored in a −80℃ ultra-low temperature freezer (Haier, Qingdao, China).

Peripheral blood concentrations of interleukin (IL)-6 and tumor necrosis factor alpha (TNF-α) were measured using the enzyme-linked immunosorbent assay (ELISA) technique (R&D Systems, Minneapolis, MN). Stress response indicators, including cortisol and epinephrine concentrations, were assessed. Serum malondialdehyde (MDA) concentrations were determined using the thiobarbituric acid (TBA) colorimetric method, while serum superoxide dismutase (SOD) concentrations were assessed using the xanthine oxidase method (hydroxylamine method).

### Statistical analysis

Data analysis was performed using SPSS 22.0 statistical software (SPSS, Chicago, IL). Descriptive statistics, including mean and standard deviation (SD), were used for continuous variables. Between-group comparisons were conducted using the *t*-test for normally distributed data, and the Mann-Whitney U test for non-normally distributed data. Within-group comparisons were assessed through repeated measures analysis of variance (ANOVA), with sphericity assumed and significance determined at a threshold of *p* < 0.05. The Mauchly's sphericity test was corrected using the Greenhouse-Geisser method for within-subject effects. Categorical data were analyzed using the chi-squared test, with a significance level set at *α* = 0.05. A *p*-value of less than 0.05 indicated statistically significant differences.”

## Results

### Preoperative characteristics of infants

In total, 116 infants were analyzed. Table 1 presents the preoperative characteristics of infants diagnosed with UPJO who underwent laparoscopic pyeloplasty, categorized by the level of CO_2_ pneumoperitoneum pressure (low and high). The mean age of infants in the LPP group was 5.63 months with a standard deviation of 1.27, while the HPP group had a mean age of 5.34 months with a standard deviation of 1.46. The mean body mass index (BMI) for the LPP group was 16.91 kg/m^2^ with a standard deviation of 2.53, and the HPP group had a mean BMI of 17.15 kg/m^2^ with a standard deviation of 2.21. In the LPP group, 45 infants (77.6%) were boys and 13 (22.4%) were girls. In the HPP group, 42 infants (72.4%) were boys and 16 (27.6%) were girls. Among patients in the LPP group, 41 infants (70.7%) had UPJO on the left side, and 17 infants (29.3%) had it on the right side. In the HPP group, 46 infants (79.3%) had left-sided UPJO, and 12 infants (20.7%) had right-sided UPJO. Within the LPP group, 47 infants (81%) had moderate UPJO, while 11 infants (19%) had severe UPJO. In the HPP group, 50 infants (86.2%) had moderate UPJO, and 8 infants (13.8%) had severe UPJO. In summary, the observed differences in age, BMI, gender distribution, side of UPJO, and severity of UPJO between the LPP and HPP groups were not statistically significant.

**Table 1:** Preoperative characteristics of infants with ureteropelvic junction obstruction (UPJO) who received the laparoscopic pyeloplasty under low (5 mmHg) and high (8 mmHg) CO_2_ pneumoperitoneum pressures.

Characteristics	LPP (*n* = 58)	HPP (*n* = 58)	*p* value
Age (month)	5.63 ± 1.27	5.34 ± 1.46	0.318
Body mass index (kg/m^2^)	16.91 ± 2.53	17.15 ± 2.21	0.401
Gender			
Boy	45 (77.6%)	42 (72.4%)	0.669
Girl	13 (22.4%)	16 (27.6%)	
Side of UPJO			
Left	41 (70.7%)	46 (79.3%)	0.391
Right	17 (29.3%)	12 (20.7%)	
Severity of UPJO			
Moderate	47 (81%)	50 (86.2%)	0.617
Severe	11 (19%)	8 (13.8%)	

The data are presented as mean ± SD or *n* (percentage). The comparisons of data were done by Mann-Whitney test or Fisher's exact test.

### Comparison of surgical indicators between the two groups of children

Table 2 provides a comparison of key surgical parameters among infants with UPJO who underwent laparoscopic pyeloplasty with varying CO_2_ pneumoperitoneum pressures. LPP group exhibited a mean blood loss of 29.72 ml with a standard deviation of 5.91, while the HPP group had a mean blood loss of 30.56 ml with a standard deviation of 6.09. In the LPP group, the mean operation time was 107.24 min with a standard deviation of 23.86. The HPP group had a mean operation time of 110.71 min with a standard deviation of 24.15. The LPP group showed a mean hospitalization time of 8.67 days with a standard deviation of 2.15. In the HPP group, the mean hospitalization time was 8.19 days with a standard deviation of 2.38. The comparison of surgical outcomes, encompassing blood loss, operation time, and hospitalization time, between infants undergoing laparoscopic pyeloplasty with low and high CO_2_ pneumoperitoneum pressures did not reveal statistically significant differences in any of the evaluated parameters.

**Table 2:** Comparisons of blood loss, operation time and hospitalization time between infants with ureteropelvic junction obstruction (UPJO) who received the laparoscopic pyeloplasty under low (5 mmHg) or high (8 mmHg) CO_2_ pneumoperitoneum pressures.

Items	LPP (*n* = 58)	HPP (*n* = 58)	*p* value
Blood loss (ml)	29.72 ± 5.91	30.56 ± 6.09	0.194
Operation time (min)	107.24 ± 23.86	110.71 ± 24.15	0.208
Hospitalization time (day)	8.67 ± 2.15	8.19 ± 2.38	0.372

The data are presented as mean ± SD. The comparisons of data were done by Mann-Whitney test.

### Comparisons of postoperative complications between the two groups of the infants

Postoperative complications among infants who underwent laparoscopic pyeloplasty for were compared in Table 3. In the LPP group, one case (1.7%) experienced an incision infection. In the HPP group, no incision infections were reported. Two cases (3.4%) in the LPP group and one case (1.7%) in the HPP group experienced urinary extravasation. One case (1.7%) in both the LPP and HPP groups developed fever postoperatively. In the LPP group, one case (1.7%) experienced hematuresis, while two cases (3.4%) in the HPP group had this complication. The total number of complications, including all categories mentioned above, was 5 cases (8.6%) in the LPP group and 4 cases (6.9%) in the HPP group. In summary, the comparison of postoperative complications between infants undergoing laparoscopic pyeloplasty with low and high CO_2_ pneumoperitoneum pressures did not show statistically significant differences in the incidence of any of the evaluated complications.

**Table 3:** Comparisons of postoperative complications between infants with ureteropelvic junction obstruction (UPJO) who received the laparoscopic pyeloplasty under low (5 mmHg) or high (8 mmHg) CO_2_ pneumoperitoneum pressures.

Items	LPP (*n* = 58)	HPP (*n* = 58)	*p* value
Incision infection	1 (1.7%)	0 (0%)	> 0.999
Urinary extravasation	2 (3.4%)	1 (1.7%)	> 0.999
Fever	1 (1.7%)	1 (1.7%)	> 0.999
Hematuresis	1 (1.7%)	2 (3.4%)	> 0.999
Total	5 (8.6%)	4 (6.9%)	> 0.999

The data are presented as *n* (percentage). The comparisons of data were done by Fisher's exact test.

### The acid-base balance parameters among infants

Table 4 presents a comparison of acid-base balance parameters among infants undergoing laparoscopic pyeloplasty for UPJO at different CO_2_ pneumoperitoneum pressures. At 5 min pre-pneumoperitoneum, the mean pH for the LPP group was 7.42 with a standard deviation of 0.08. In the HPP group, the mean pH was 7.43 with a standard deviation of 0.09. pH levels showed no significant difference between the two groups at this time point. After 30 min of pneumoperitoneum, the pH for the LPP group decreased to 7.36 with a standard deviation of 0.07, and for the HPP group, it decreased to 7.21 with a standard deviation of 0.06. Both groups exhibited a statistically significant decrease in pH values compared to their respective pH levels at 5 min pre-pneumoperitoneum. At 5 min pre-pneumoperitoneum, the mean PaCO_2_ for the LPP group was 40.14 mmHg with a standard deviation of 3.71, while the HPP group had a mean PaCO_2_ of 39.82 mmHg with a standard deviation of 3.88. PaCO_2_ levels did not significantly differ between the groups at this time point. However, at 30 min post-pneumoperitoneum, both groups experienced significant increases in PaCO_2_ levels compared to their respective pre-pneumoperitoneum values. At all time points, PaO_2_ levels did not display significant differences between the two groups. Similar trends were observed in both ABE and SBE values, with no significant differences between groups at the pre-pneumoperitoneum time point. However, after 30 min of pneumoperitoneum, the HPP group showed significantly lower ABE and SBE values compared to their pre-pneumoperitoneum values. The results presented above indicate that both LPP (5 mmHg) and HPP (8 mmHg) can lead to respiratory acidosis in infants. Furthermore, it is evident that an CO_2_ pneumoperitoneum pressure of 8 mmHg has a more pronounced impact on the acid-base balance in pediatric patients compared to the pressure of 5 mmHg.

**Table 4:** Comparisons of acid-base balance between infants with ureteropelvic junction obstruction (UPJO) who received the laparoscopic pyeloplasty under low (5 mmHg) or high (8 mmHg) CO_2_ pneumoperitoneum pressures.

Parameters	Group	5 min pre- pneumoperitoneum	5 min post- pneumoperitoneum	30 min post- pneumoperitoneum	30 min after pneumoperitoneum release
PH	LPP (*n* = 58)	7.42 ± 0.08	7.41 ± 0.07	7.36 ± 0.07	7.40 ± 0.08
	HPP (*n* = 58)	7.43 ± 0.09	7.36 ± 0.06	7.21 ± 0.06*^,#^	7.31 ± 0.07*^,#^
PaCO_2_ (mmHg)	LPP (*n* = 58)	40.14 ± 3.71	42.98 ± 3.67	49.71 ± 4.09^#^	40.04 ± 3.49
	HPP (*n* = 58)	39.82 ± 3.88	46.02 ± 3.81	58.82 ± 4.25*^,#^	49.72 ± 3.78*^,#^
PaO_2_ (mmHg)	LPP (*n* = 58)	94.17 ± 3.91	93.82 ± 3.72	89.68 ± 4.02	92.46 ± 3.97
	HPP (*n* = 58)	94.62 ± 3.84	92.19 ± 3.95	91.39 ± 3.84	93.77 ± 4.02
ABE (mM)	LPP (*n* = 58)	1.98 ± 0.21	2.06 ± 0.23	2.28 ± 0.19	2.19 ± 0.26
	HPP (*n* = 58)	1.91 ± 0.24	2.28 ± 0.31	2.81 ± 0.36*^,#^	2.31 ± 0.33
SBE (mM)	LPP (*n* = 58)	2.06 ± 0.25	2.21 ± 0.28	2.37 ± 0.31	2.42 ± 0.41
	HPP (*n* = 58)	2.11 ± 0.28	2.38 ± 0.33	2.95 ± 0.39*^,#^	2.46 ± 0.39

The data are presented as mean ± SD.

* *p* < 0.05 between two groups at the same time.

^#^ *p* < 0.05 compared to 5 min pre- pneumoperitoneum in the same group.

### Comparison of various stress indexes, inflammatory cytokines, and oxidative stress markers in infants

As shown in Table 5, before the operation, the mean cortisol level in the LPP group was 155.42 nmol/L with a standard deviation of 31.15. At the end of the operation, it increased to 348.92 nmol/L, and at 24 h post-operation, it decreased to 189.73 nmol/L. In the HPP group, the mean cortisol level before the operation was 150.21 nmol/L, and it significantly increased to 462.81 nmol/L at the end of the operation and decreased to 218.19 nmol/L at 24 h post-operation. Similar trends were observed for norepinephrine levels, with both groups experiencing a significant increase in norepinephrine levels at the end of the operation and a subsequent decrease 24 h after the operation. Both IL-6 and TNF-α levels showed an increase at the end of the operation in both groups, with the HPP group experiencing higher levels compared to the LPP group. Both cytokines demonstrated a decrease 24 h after the operation. MDA levels increased at the end of the operation and then slightly decreased 24 h after the operation in both groups. SOD levels decreased at the end of the operation, and the decrease was more significant in the HPP group compared to the LPP group. The presented results collectively indicate that both LPP (5 mmHg) and HPP (8 mmHg) can lead to a certain degree of stress response, elevation in inflammatory cytokines, and oxidative stress in infants. Moreover, it is evident that a CO_2_ pneumoperitoneum pressure of 8 mmHg has a more pronounced and significant impact on stress response, elevation in inflammatory cytokines, and oxidative stress in pediatric patients compared to the pressure of 5 mmHg.

**Table 5:** Comparisons of stress indexes, inflammatory cytokines and oxidative stresses between infants with ureteropelvic junction obstruction (UPJO) who received the laparoscopic pyeloplasty under low (5 mmHg) or high (8 mmHg) CO_2_ pneumoperitoneum pressures.

Parameters	Group	Before operation	End of the operation	24 h after operation
Cortisol (nmol/L)	LPP (*n* = 58)	155.42 ± 31.15	348.92 ± 42.28^#^	189.73 ± 34.89
	HPP (*n* = 58)	150.21 ± 32.64	462.81 ± 54.76*^,#^	218.19 ± 38.44
Norepinephrine (pmol/L)	LPP (*n* = 58)	894.77 ± 159.71	1,932.08 ± 266.71^#^	979.05 ± 172.33
	HPP (*n* = 58)	906.26 ± 152.63	2,538.27 ± 308.86*^,#^	1,125.85 ± 190.04
IL-6 (pg/ml)	LPP (*n* = 58)	27.74 ± 5.17	39.82 ± 9.81^#^	31.73 ± 6.82
	HPP (*n* = 58)	29.81 ± 6.36	52.15 ± 11.36*^,#^	40.23 ± 7.05*^,#^
TNF-α (pg/ml)	LPP (*n* = 58)	21.15 ± 4.32	33.83 ± 5.89^#^	28.71 ± 5.62^#^
	HPP (*n* = 58)	19.96 ± 4.49	45.21 ± 8.72*^,#^	37.77 ± 7.83*^,#^
MDA (nM)	LPP (*n* = 58)	4.37 ± 0.93	5.41 ± 1.03^#^	4.62 ± 0.98
	HPP (*n* = 58)	4.41 ± 1.06	6.77 ± 1.12*^,#^	4.94 ± 1.09
SOD (U/ml)	LPP (*n* = 58)	73.26 ± 11.15	65.83 ± 12.09	69.94 ± 12.27
	HPP (*n* = 58)	75.02 ± 12.81	51.81 ± 10.74*^,#^	62.23 ± 11.36^#^

The data are presented as mean ± SD.

* *p* < 0.05 between two groups at the same time.

^#^ *p* < 0.05 compared to 5 min pre- pneumoperitoneum in the same group.

## Discussion

UPJO represents a significant urological condition characterized by a constriction or partial blockage at the junction of the renal pelvis and ureter (2). This obstruction can lead to diverse clinical manifestations, necessitating prompt medical intervention to preserve renal function and mitigate complications (19). Laparoscopic pyeloplasty has emerged as a pivotal surgical approach for UPJO, offering enhanced precision and reduced patient morbidity (20). This procedure's minimally invasive nature translates to diminished tissue trauma, expedited recovery, and reduced postoperative complications (21, 22).

CO_2_ pneumoperitoneum is a requisite for laparoscopic surgeries, with CO_2_ gas being the most commonly employed insufflation agent (23, 24). While higher insufflation pressures enhance surgical visualization, they concurrently impose physiological stress on the body (25). Previous investigations into the effects of CO_2_ insufflation have predominantly focused on adults and older children, leaving a gap in knowledge concerning the effects on infants (26). Given the less developed physiological regulatory systems in infants, studying the impact of CO_2_ insufflation on their physiology is of profound clinical significance.

This study delved into the effects of different CO_2_ pneumoperitoneum pressures during laparoscopic pyeloplasty on various parameters in infants with UPJO. The randomized controlled trial design ensured robust comparisons. The study provides a comprehensive overview of the preoperative characteristics of the included infants. Despite some differences in mean age, BMI, gender distribution, side of UPJO, and severity of UPJO between the LPP and HPP groups, these variations were not statistically significant. These results imply that the allocation of patients into the two pressure groups was relatively balanced in terms of baseline characteristics, reducing the potential for confounding factors.

The comparison of surgical indicators such as blood loss, operation time, and hospitalization time is of clinical relevance. The study demonstrates that there were no statistically significant differences in these indicators between the LPP and HPP groups. This suggests that variations in CO_2_ pneumoperitoneum pressures did not exert a significant impact on the surgical outcomes evaluated in this study. The consistency in these parameters across the pressure groups implies that both pressures could be considered safe and feasible for the laparoscopic pyeloplasty procedure in this specific patient population.

Postoperative complications are critical factors affecting patient recovery and clinical outcomes. The study's findings indicate that there were no statistically significant differences in the incidence of various complications, including incision infection, urinary extravasation, fever, and hematuria, between the LPP and HPP groups. These results suggest that the choice of CO_2_ pneumoperitoneum pressure might not be a primary determinant of the occurrence of these complications in infants undergoing laparoscopic pyeloplasty for UPJO.

The assessment of acid-base balance parameters provides insights into the potential physiological effects of CO_2_ pneumoperitoneum pressures on infants. The study demonstrates that both LPP (5 mmHg) and HPP (8 mmHg) pressures can lead to a decrease in pH values after 30 min of pneumoperitoneum compared to pre-pneumoperitoneum levels. Additionally, the HPP group experiences more significant reductions in ABE and SBE after 30 min of pneumoperitoneum compared to the LPP group. These findings indicate that higher CO_2_ pneumoperitoneum pressures might induce more pronounced respiratory acidosis and metabolic disturbances, especially when sustained over a certain duration.

The study's assessment of stress response, inflammatory cytokines, and oxidative stress markers sheds light on the physiological responses induced by varying CO_2_ pneumoperitoneum pressures. Notably, both LPP and HPP groups experienced an increase in stress markers, inflammatory cytokines, and oxidative stress markers at the end of the operation, with the HPP group showing more substantial increases. This suggests that both pressures trigger physiological responses, but the higher pressure of 8 mmHg induces a more prominent stress reaction and inflammation.

While this study provides valuable insights into the effects of different CO_2_ pneumoperitoneum pressures on infants undergoing laparoscopic pyeloplasty for UPJO, several limitations should be acknowledged. (1) The relatively small sample size of 116 patients, though adequately powered for the conducted analyses, might limit the generalizability of the findings. A larger, multicenter study involving a broader range of patient demographics would enhance the external validity of the results. (2) Conducted within a single medical institution, this study's findings might not be representative of the broader population due to potential institutional variations in surgical techniques, patient care, and anesthesia protocols. (3) The study's focus on short-term outcomes up to 24 h postoperatively provides insight into immediate effects but precludes a comprehensive assessment of longer-term implications. A longer follow-up period would be required to evaluate the persistence and evolution of the observed physiological changes. (4) This study exclusively focused on variations in CO_2_ pneumoperitoneum pressures while holding other parameters constant. The effects of altering other insufflation parameters, such as gas flow rate or duration, were not explored and could have contributed to a comprehensive understanding of their combined effects. (5) Only the levels of IL-6 and TNF-α were examined in the current study. To fully evaluate the inflammatory levels, other pro- and anti-inflammatory cytokines, such as IL-1β and IL-10, should be considered in the future work.

## Conclusions

In conclusion, this study sheds light on the distinct effects of CO_2_ pneumoperitoneum pressures on various physiological parameters in infants undergoing laparoscopic pyeloplasty for UPJO. These findings emphasize the significance of carefully selecting insufflation pressures, particularly in the delicate pediatric population, to minimize physiological disturbances and enhance patient outcomes. This study contributes valuable insights to the field of pediatric laparoscopic surgery, guiding clinicians towards optimized perioperative care strategies.

## Data Availability

The raw data supporting the conclusions of this article will be made available by the authors, without undue reservation.
